# Modified QuEChERS Extraction and HPLC-MS/MS for Simultaneous Determination of 155 Pesticide Residues in Rice (*Oryza sativa* L.)

**DOI:** 10.3390/foods9010018

**Published:** 2019-12-24

**Authors:** Maria Graça Melo, Ana Carqueijo, Andreia Freitas, Jorge Barbosa, Ana Sanches Silva

**Affiliations:** 1 National Institute for Agricultural and Veterinary Research (INIAV), Rua dos Lágidos, Lugar da Madalena, 4485-655 Vila do Conde, Portugal; graca.melo@iniav.pt (M.G.M.); ana.carqueijo@iniav.pt (A.C.); andreia.freitas@iniav.pt (A.F.); jorge.barbosa@iniav.pt (J.B.); 2 REQUIMTE/ LAQV, Pharmacy Faculty, University of Coimbra, Azinhaga de Santa Comba, 3000-548 Coimbra, Portugal; 3 Center for Study in Animal Science (CECA), ICETA, University of Oporto, 4051-401 Oporto, Portugal

**Keywords:** cereals, high-performance liquid chromatography-tandem mass spectrometry, pesticides, quick, easy, cheap, effective, rugged, and safe (QuEChERS) method, rice, validation

## Abstract

Rice (*Oryza sativa* L.) is the staple food of more than half of the world’s population. The main factors affecting the quality of rice include grain length, texture, stickiness, flavor, and aroma. Pesticides are intended for the protection of plant products from weeds, fungi, or insects. However, pesticides also result in negative effects such as environment disturbances, pest resistance and toxicity to both users and food consumers. The aim of this study was to conduct validation experiments of a method for the determination of multi-pesticides in rice, a model food of other cereals. A quick, easy, cheap, effective, rugged, and safe (QuEChERS) method was used for the extraction of pesticide residues from rice followed by high-performance liquid chromatography-tandem mass spectrometry (HPLC-MS/MS) with a triple quadrupole instrument using electrospray ionization. The analytical method has chromatography-tandem according to SANTE/11813/2017. The limit of quantification was 5 μg/kg. Recoveries for the 155 analyzed pesticides ranged between 77.1% for pirimiphos-ethyl and 111.5% for flutriafol and they were determined at 3 spiking levels. The proposed method was demonstrated to be quick, simple, precise, and accurate and allowed for evaluating the compliance of cereals samples with legislated maximum residue levels of pesticides in the European Union.

## 1. Introduction

Rice is the staple food of more than half of the world’s population [1]. There are several types of rice that meet different consumer preferences. The main factors affecting the quality of rice are grain length (a higher proportion of broken grains decreases the economic value of rice), texture, stickiness, flavor, and aroma. The nutritional composition of rice varies among different types of rice but in general high-performance, it is rich in macro and micronutrients and an excellent source of complex carbohydrates.

Cultivated (Asian) rice (*Oryza sativa* L.) includes the long-grain variety group (*indica*) and the short grain variety group (*japonica* or *sinica*) [2]. The length/width ratio of the *indica* variety is 4 to 5 while in *japonica* it is around 2 [3]. Basmati and jasmine rice are examples of *indica* rice. *Japonica* rice is the sticky, moist, bright, white rice generally used in sushi, Mediterranean, and Asian dishes, which require more stickiness [2]. Post-harvest processing of any variety of rice can produce either white or brown rice. This affects texture, flavor, and nutritive value.

The demands of an increasing population for safe and high-quality food products has dictated the use of intensified agriculture and the increasing use of agrochemicals to control weeds/pests and damages caused by the insect or fungi population [4]. Although the efforts to reduce or find alternatives are in fast development, the use of pesticides is still a reality and in fact, they are crucial to avoid food loss. However, pesticides also result in environmental disturbances (air, soil, water), pest resistance, pest resurgence, acute and chronic effects to non-target organisms in the agroecosystems and toxicity to both users and food consumers [4].

Therefore, the control of pesticide residues in food is of utmost importance and in the European Union, it is supported by legislation, to ensure the safety of the population as well as national and international trade. The use of pesticides in the EU is established in the Regulation (EC) No. 396/2005 and amendments [5] and Regulation (EU) No. 2018/62 [6].

The European Commission has set harmonized maximum residue levels (MRL) in Regulation 396/2005 [5] to prevent different Member States from having different MRL for the same pesticide in the same product. Thus, multi-residue methodologies capable of simultaneously determining a large number of pesticides are required.

In the analysis of pesticides, different extraction procedures have been used to efficiently separate the analysts of interest from the food matrix. Conventional methods used to determine pesticides are time-consuming and complex. The recent extraction procedure called QuEChERS (Quick, Easy, Cheap, Effective, Rugged, and Safe) was developed by Anastassiades et al. [7] and it is based on acetonitrile extraction followed by partition and cleaning up steps by dispersive solid-phase extraction (d-SPE). Initially, this method was developed to be applied to food matrices with high water (>75%) and low-fat content [8]. However, after some adjustments, it was proven to be possible to apply it to dry and fatty food. In this line, the QuEChERS-based methods present several advantages besides efficiency, such as simplicity, good accuracy, short analysis time, amenable to high throughput, high recovery for compounds with a wide range of polarities, use of smaller amounts of organic solvent and no use of chlorinated solvents [9]. Therefore, several studies have used QuEChERS to analyze pesticides in rice, as it is summarized in Table 1.

Recently other newly developed sample preparation methods have been used for the analysis of pesticides in food samples, such as carbonaceous nanomaterial supported solid-phase extraction. Some of the carbonaceous nanosorbents already reported include graphene derivatives modified by combination with silica, amines, polymers, and/or magnetic nanoparticles [16]. In what concerns the analytical techniques, gas chromatography (GC) coupled with nitrogen-phosphorus detection (NPD) [17], electron capture detection (ECD) [18], or mass spectrometry (MS) [19] have been widely used. However, GC is not appropriate for non-volatile molecules or compounds thermally unstable such as benzimidazoles and carbamates. Therefore, HPLC coupled with MS/MS is a tool that enables the determination of multiple pesticide residues minimizing the matrix components interferences. The only drawbacks are related to molecules that produce the fragment of identical mass, which is not common [13].

The aim of this study was to conduct validation experiments of a method for the determination of multi-pesticides fortified between 5 and 50 μg/kg in rice, a model food of other cereals, and cereal-based food. Validation followed the guidance document SANTE/11813/2017 [20].

## 2. Materials and Methods

### 2.1. Chemicals and Reagents

Methanol, acetonitrile (both HPLC gradient grade), toluene, acetone, ethanol, ethyl acetate, n-hexane, and formic acid were purchased from Merck (Darmstadt, Germany). Water was purified by Milli-Q plus system from Millipore (Molsheim, France). Trisodium citrate dihydrate and disodium hydrogencitrate sesquihydrate were purchased from Sigma-Aldrich (Madrid, Spain) while NaCl was purchased from Fischer. Primary secondary amine bonded silica (PSA) was acquired from Supelco (Supelclean™, Bellefonte, PA, USA). Anhydrous magnesium sulfate was purchased from Fluka. Ammonium formate was acquired from VWR. Pesticide standards and internal standard (triphenylphosphate-TPP and dinitrocarbanilide or 1,3-bis(4-nitrophenyl)urea-DNC) were purchased from Sigma–Aldrich (Madrid, Spain) and were dissolved in toluene, acetone, ethanol, ethyl acetate, methanol, n-hexane, or acetonitrile, depending on the solubility of the compound, at a concentration of 5 mg/L. These stock solutions were subsequently used to prepare different working solutions for calibrations. Working solutions were prepared in acetonitrile. All standard solutions were stored in amber vials in the dark at −20 °C, for at least 3 years [20], and before use, they were kept at room temperature for 15 min. 

### 2.2. Samples and Sampling Procedure

Twenty-five samples of rice were purchased from a local supermarket (Oporto, Portugal) in the summer of 2019 for quantification of multi-pesticide residues. Rice belongs to the following types: 5 long-grain rice samples, 10 samples of medium-grain rice of the Portuguese variety Carolino, 5 samples of Basmati rice and 5 samples of parboiled rice. Each laboratory sample (1 kg) was homogenized by grinding (Retsch rotor mill SK 300 with a sieve of trapezoid holes of 1.00 mm) and the flours were mixed thoroughly to assure complete homogenization. Each sample was placed in separate sample collection tubes (50 g approx.) and preserved at −20 °C until analysis.

### 2.3. Extraction Procedure

The procedure involved the extraction of 10 g rice with 10 mL acetonitrile after mixing the sample with cold water (20 g). Subsequently, a liquid–liquid partitioning step performed by adding a mixture of MgSO_4_, NaCl, trisodium citrate dihydrate, and disodium hydrogen citrate sesquihydrate (4:10:1:0.5 *w/w/w/w*). After centrifugation, 6 mL of the extract was added into a tube containing 150 mg primary secondary amine (PSA) sorbent plus 0.9 g anhydrous MgSO_4_, which corresponds to a cleanup step, called dispersive solid-phase extraction. After a second shaking and centrifugation step, 220 μL acetonitrile is added to 1 mL of the extract. Then the internal standards solution was added to the extract before being analyzed by high-performance liquid chromatography-tandem mass spectrometry (HPLC-MS/MS) with a triple quadrupole instrument using electrospray ionization (ESI). The IS is added just before LC-MS analysis to correct for instrumental variations.

### 2.4. HPLC–MS/MS Parameters

The analytical method has been validated according to SANTE/11813/2017 [20]. 

Detection and quantification were performed with a UHPLC Nexera X2 (Shimadzu, Kyoto, Japan) coupled with QTRAP 5500+ MS/MS detector (AB SCIEX, Foster City, CA, USA) equipped with an electrospray ionization (ESI) source working simultaneously in both positive and negative modes (ESI+ and ESI−). In terms of chromatographic conditions, a column Synergi 4 µm Fusion-RP 80A 50 × 2 mm (Phenomenex, Torrance, CA, USA) was used and kept at 35 °C, the autosampler was maintained at 10 °C to refrigerate the samples and a volume of 10 μL of sample extract was injected in the column. The mobile phase consisted of the gradient reported in Table 2, using 0.1% formic acid in ultrapure water as mobile phase [A] and formic acid 0.1% in methanol as mobile phase [B] with a flow rate of 0.25 mL/min. 

The total run time was 18 min. In terms of mass spectrometry the acquisition was performed in MRM mode from 100 to 750 Da using the Analyst^®^ TF (SCIEX, Foster City, CA, USA) software (SCIEX, Foster City, CA, USA) and with the following settings: ion spray voltage of 4500 V; source temperature 600 °C; curtain gas (CUR) at 35 psi; gas 1 and gas 2 at 40 and 60 psi, respectively. 

Parameters for the determination of pesticide residues in rice, by MS/MS in ESI+ and in ESI− mode, are presented in Tables S1 and S2, (Supplementary Materials), respectively. Data acquisition in the multiple reaction monitoring (MRM) mode was optimized after direct infusion, into the detector, of each individual standard solution of 1 μg/mL Thus, two ion transitions were selected for each compound, a quantifier and a qualifier MRM.

### 2.5. Identification and Quantification of Pesticide Residues

The identification and data processing of pesticide residues were made through the MultiQuant^TM^ software (SCIEX, Foster City, CA, USA).

In terms of identification criteria, two parameters were used, in accordance with the SANTE (2017) [20]: retention time (RT) with a tolerance of ±0.1 min in relation to the RT of the analyte in calibration standard (may need to be matrix-matched) and ion ratio tolerance below 30%. The use of an internal standard in mass spectrometry methodologies is advisable to access possible variations during the analytical process. 

Equation (1): Deviation of RRT,

(1) $$\Delta RRT\;=(RT_{sample}-RT_{mean\;calibration}),$$

where *RT_sample_* is the retention time of the analyte in a sample and *RT_mean calibration_* corresponds to the mean of retention time obtained, for the same analyte, in a set of calibrations (may need to be matrix-matched).

The ion ratio is determined as the ratio between the areas obtained for both ion transitions of each analyte.

Equation (2): Ion ratio (IR, %),

(2) $$IR=\left(\frac{A_{\;ion\;with\;lowest\;intensity}}{A_{ion\;with\;highest\;intensity}}\right)\times 100.$$

In Equation (2), *A_ion with lowest intensity_* corresponds to the area of the ion with the lowest intensity and the *A_ion with highest intensity_* to the area of the ion with the highest intensity.

Equation (3): Deviation of IR (ΔIR, %),

(3) $$\Delta IR=\;\frac{IR_{sample}-IR_{mean\;calibration}}{IR_{mean\;calibration}}\;\times 100,$$

in which *IR_sample_* corresponds to the ion ratio obtained for a target compound present in a sample and *IR_mean calibration_* refers to the mean ion ration obtained for a batch of calibration of the same analyte.

The positive identification is achieved if both criteria is fulfilled (ΔRRT < 0.1 min and ΔIR < 30%) (Equations (2) and (3)).

### 2.6. Validation of HPLC–MS/MS Method for Multi-Pesticides Residues

The validation of the method was carried out by the evaluation of the following parameters: concentration range, linearity, the limit of quantification (LOQ), precision (repeatability and intra-laboratory reproducibility) and accuracy (using recovery assays). Furthermore, the expanded uncertainty was also calculated at the LOQ level in accordance with the equations presented below.

Equation (4): Combined uncertainty (*U_C_*),

(4) $$U_{C}=y\times \sqrt{{\left(U_{accuracy}\right)}^{2}+{\left(U_{precision}\right)}^{2}},$$

where *y* is the concentration for which the uncertainty is being measured, in this case for the LOQ, *U_accuracy_* is the uncertainty associated with accuracy and *U_precision_* is the uncertainty associated with precision. 

Equation (5): Expanded uncertainty (U),

(5) $$U=k\times U_{C}.$$

For a level of confidence of 95%, k should be considered as 2 (SANTE/11813/2017) [20].

The limit of quantification corresponds to the lowest calibration level (LCL), which is lower than the reporting limit (RL). For the determination of repeatability (RSD_r_) and intra-laboratory reproducibility (RSD_R_), blank samples of rice were spiked at 3 different levels (*n* = 5). In the case of RSD_R_ extraction was carried out in 3 different days by 3 different operators. The accuracy of the method was evaluated using recovery assays.

#### 2.6.1. Spiking Experiment

To determine the recovery of the target analytes, spiking experiments were performed. Calibration standards were prepared by spiking blank sample of rice (10 g) with 3 different concentrations 5, 10, and 50 μg/kg, of a multi-pesticide standard solution prepared in acetonitrile (*v/v*), thoroughly mixed, and kept at ambient temperature in the dark for 30 min. Afterward, extraction was performed as described in Section 2.3.

#### 2.6.2. The Matrix Effect

Matrix effect was evaluated according to SANTE/11813/2017 [20] comparing the response of the pesticides obtained in the standard solution with the response in the fortified rice sample. The ratio between the slope obtained from the matrix-matched calibration curve and the curve obtained by external calibration was calculated for all the pesticide residues. Assays were calculated in triplicate.

## 3. Results

### 3.1. Optimization of the Method Conditions

A modified QuEChERS method was used for the extraction of pesticide residues from rice. The procedure involved the extraction of 10 g rice with 10 mL acetonitrile after mixing the sample with water (20 g) and it was left to stand for about one hour. Different amounts of cold water were tested to assure the required rice swelling. The best recovery results (data not shown) were achieved with 20 ml. Hou et al. [1], used 10 mL water to swelling 5 g sample (ratio sample:water 1:2). After the addition of acetonitrile, some authors put the extracts in the refrigerator. For instance, Hou et al. [1] left the extracts 30 min in the refrigerator while in our method the solution was left to stand one hour. According to this author, this step could counteract the heat that is generated by the salts and that can deform the Falcon tubes. Subsequently, a liquid–liquid partitioning step was performed by adding a mixture of MgSO_4_, NaCl, trisodium citrate dihydrate, and disodium hydrogen citrate sesquihydrate (4:10:1:0.5 *w/w/w/w*). After centrifugation, the extract was decanted into a tube containing 150 mg primary secondary amine (PSA) sorbent plus 0.9 g anhydrous MgSO_4_, which corresponds to a cleanup step called dispersive solid-phase extraction. PSA is used because being a weak anion exchange can remove organic acids, some sugars, and fatty acids [12]. Hou et al. [1] tested different amounts of PSA (25–150 mg/mL extract) and concluded the best to reduce the content of the extract on fatty acids was 75 mg PSA/mL extract, therefore it used 375 mg PSA in the extraction procedure. In the present method, 1.05 g PSA mixture (150 mg PSA sorbent plus 0.9 g anhydrous MgSO_4_) was used for 6 mL of extract which corresponds to 175 mg/mL extract.

After a second shaking and centrifugation step, 1 mL of extract was added to 220 μL acetonitrile. Then the internal standards solution was added to the extract just before being analyzed by HPLC-MS/MS with a triple quadrupole instrument using ESI. 

Most of the pesticide residues were analyzed in ESI+ (152 of the total of 155 pesticides), just fludioxonil, fipronil, and methoxyfenozide were analyzed in the ESI−mode (Tables S1 and S2). The IS used in the present method for ESI+ mode was TPP but other studies used different IS like chlophrifos-d10 [1]. For the ESI−method, the internal standard was DNC.

**Figure 1:**
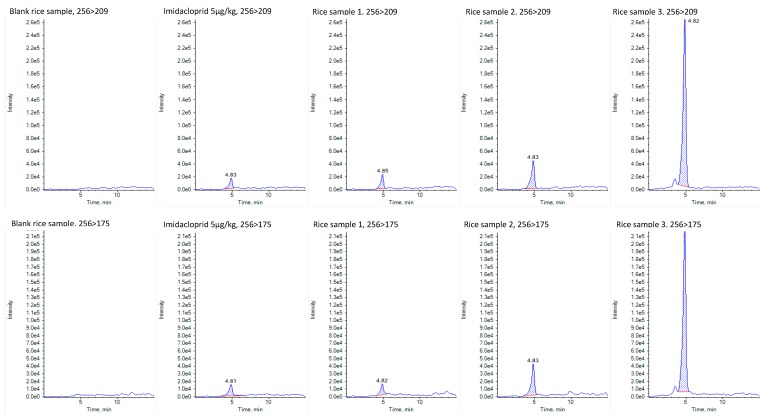
MS/MS chromatogram of a blank rice sample, imidacloprid standard (5 μg/kg), rice sample 1, rice sample 2, and rice sample 3, showing both transitions of imidacloprid (256 > 209 and 256 > 175).

Separation of the 155 pesticide residues was achieved in an 18 min chromatographic run (Figure 1). Most of these 155 pesticides were insecticides (80), fungicides (60) or herbicides (9) (Tables S1 and S2, Supplementary Materials). The method was validated according to the criteria defined by SANTE/11813/2017 [20], which establishes the validation parameters for the official control of the pesticides in cereals in the EU. Identification criteria were described in Section 2.5, and were always evaluated. In the experiments carried out for validation purposes, ΔRRT deviation was always lower than 0.1 min. Moreover, ion ratio tolerance always met the defined criterion which was lower than 30%.

### 3.2. Validation of the Method

Linearity was evaluated by both calibration curves and matrix-matched calibration curves in different ranges for different pesticide residues (see Table 3). The linear range of the calibration curves ranged between 5–50 or 5–60 μg/L, depending on the pesticide. The limit of quantification was 5 μg/kg. The determination coefficient varied between 0.9691–0.998, indicating suitability for pesticide quantification. Table 3 shows the results of linearity, precision, and accuracy (determined through recovery studies) for the different pesticide residues in a blank rice sample spiked at 3 levels. Recoveries for the 155 analyzed pesticides ranged between 77.1% for pirimiphos-ethyl and 111.5% for flutriafol and they were determined at 3 spiking levels (5, 10, and 50 μg/kg). 

The recoveries of the methods were all within the appropriated range of the SANTE/11813/2017 [20] criteria. Repeatability of the method was evaluated by the Relative Standard Deviation RSDr. RSDr was between 1.18% and 17.9 % at 5 μg/kg; 2.23% and 17.4% at 10 μg/kg; 2.79% and 18.6% at 50 μg/kg. 

Reproducibility was evaluated by the Relative Standard Deviation RSD_R_ at 3 different days of analysis, different concentration levels and with different operators and values were considered acceptable (varied between 3.20% and 17.5 %). The limit of quantification was 5 μg/kg, which is sensitive enough to meet the requirements imposed by EU regulations for the MRL of pesticide residues in cereals limit of report (10 μg/kg).

Matrix effect was inexistent for ethion, methacrifos, pencycuron, and tolclofos-methyl. However, it was found signal enhancement with a deviation higher than 20% for fipronil, methomyl, quinoxyfen, thiabendazole, and thiophanate- methyl. Regarding signal suppression, this was found with a deviation higher than 20% for flufenoxuron, lufenuron, parathion-methyl, metaflumizone, pirimicarb, and teflubenzuran. 

Expanded uncertainty was calculated according to the equations included in Section 2.6. and ranged between 10% for pencycuron and 43% for methiocarb sulfoxide. Therefore, it is concluded that the pesticide residue results do not have to be adjusted for recovery because the mean recovery is within the range of 80%–120% and the criteria of 50% expanded measurement uncertainty is fulfilled. This is in accordance with SANTE/11813/2017 [20].

Matrix effect can be caused by the co-elution of matrix components and affects the efficiency of the ionization of the analytes. The signal suppression-enhancement (SSE) was used to determine the matrix effect of the pesticides’ residues in rice. SSE was calculated as follows:

SSE(%) = (matrix-matched calibration slope/standard calibration slope) × 100.(6)

Signal enhancement was considered when SSE > 100%, inexistence of the matrix effect when SSE = 100% and signal suppression when SSE < 100%.

### 3.3. Pesticides Residues in Rice Commercial Samples

**Figure 2:**
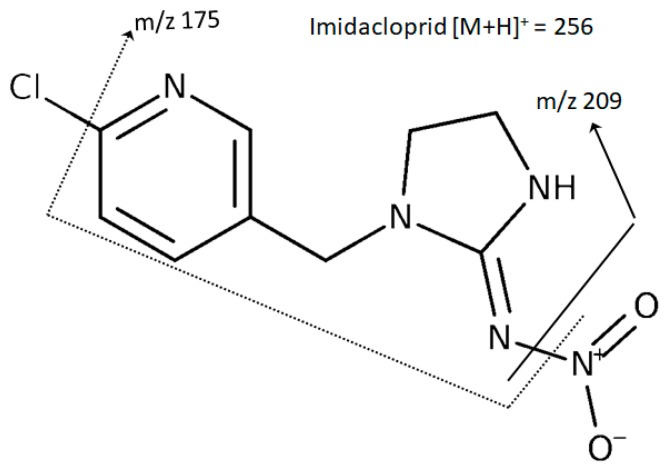
Profile of fragmentation of the insecticide imidacloprid obtained in the optimization of the HPLC-MS/MS method.

Twenty-five commercial rice samples were analyzed regarding their content in the 155 pesticide residues included in the HPLC-MS/MS methods described earlier. Rice samples were collected from July till September 2019. Samples were negative for all pesticides residues although the insecticide imidacloprid was found in 3 samples (rice sample 1: 0.0054 ± 0.0008 mg/kg, rice sample 2: 0.0125 ± 0.0005 mg/kg, and rice sample 3: 0.0658 ± 0.0018 mg/kg) (Figure 1). Sample 1 corresponds to a Basmati rice, sample 2 to medium-grain rice, and the contaminated sample 3 corresponds to parboiled rice. However, the MRL for this pesticide was 1.5 mg/kg, therefore none of the samples exceeded EU MRL for rice [21]. The two transitions of imidacloprid selected have already been selected by Carneiro et al. [22] for the determination of pesticides in bananas by modified QuEChERS and UHPLC-MS/MS analysis, although in this study, in opposite to our method, the quantification transition was 256.2 > 175.1 and the confirmation transition was 256.2 > 209.1. The ion 256 corresponds to [M+H]^+^, the ion 209 corresponds to the loss of the group nitro (−NO_2_) from the molecule and the ion 175 to the loss of both −NO_2_ and −Cl from the molecule (Figure 2).

In 1994, a study reported an HPLC method to determine imidacloprid as a new insecticide in rice and cucumber [23]. Ishii et al. [23] stated that imidacloprid was effective against a vast range of pest species (e.g., whiteflies, scales, psyllids, plant bugs, leafhoppers, and planthoppers) and also mentioned that this insecticide is an agonist of acetylcholine by binding to nicotinergic acetylcholine receptors or postsynaptic membrane. Other studies reported positive rice samples, although none of these has reported the presence of imidacloprid.

Nguyen et al. [9] has analyzed 93 varieties of rice and found just one positive with fenobucarb at a level of 0.65 mg/kg. Ahmad et al. [4] analyzed 400 rice samples regarding 4 different pesticides (Lambda-cyhalothrin, malathion, novacron, and cartap hydrochloride) and found levels between 19 and 148 mg/kg. Shakouri et al. [13] analyzed 60 rice samples for 41 pesticides and found 11 domestic samples and 1 imported sample contaminated. Rebelo et al. [14] analyzed 8 samples of rice regarding 18 herbicides and all samples were negative.

In the last 15 years, several notifications were reported through the Rapid Alert System for Food and Feed (RASFF) [24] in rice in Portugal. One arose in 2005, and it was related to the presence of phosmet (0.06; 0.05; 0.06 mg/kg) and diazinon (0.58; 0.33; 0.40 mg/kg) in rice from Portugal. Another one arose in the same year and it was related to deltamethrin (2.1 mg/kg) in rice from Guyana. In 2015, there was another notification regarding an unauthorized substance triazophos (0.04 mg/kg) in basmati rice from India. Recently another notification was related to an unauthorized substance tricyclazole (0.092 mg/kg) in parboiled and brown rice from Brazil.

## 4. Concluding Remarks

The QuEChERS method was clearly demonstrated to be quick, simple, reliable, and effective for the determination of 155 pesticide residues in rice. The proposed method is applicable for the routine analysis of pesticide residues in cereals and demonstrated to be sensitive, precise, and accurate. Moreover, it is suitable to evaluate the compliance of cereals samples with legislated maximum residue levels of pesticides in the European Union. None of the pesticide residues were found in the analyzed samples, except the insecticide imidacloprid which was found in three samples at levels above the MRL. However, appropriate and extended sampling is needed in Portugal to better evaluate the level of compliance of rice with the current legislation in force and to be possible to evaluate the probability of occurrence of a pesticide according to the rice source or rice type.

## Figures and Tables

**Table 1:** Compilation of methods to determine pesticides in rice samples.

Extraction Technique	Chromatographic Technique	Pesticides	No. of Rice Samples	LOD/LOQ	Recovery	References
Extraction with DCM and clean up with Florisil SPE column	GC-MS	40		LOD: 0.26–87 μg/kg	Most of them: 75%–120%	[10]
GPC	GC-MS	109		LOD: 1–20 ng/g	Most of them: 70%–110%	[11]
QuEChERS	GC/MS-SIM	109	93 varieties of rice and 1 positive (fenobucarb 0.65 mg/kg)	0.002–0.05 mg/kg	75%–115%	[9]
QuEChERS	UHPLC-ESI-MS/MS	13 phenoxy acid herbicides		LOD: 0.0005–0.005 mg/kg	45%–104%	[12]
Soxhlet extraction with acetone and ethyl acetate (1:2)	GC-FID	4 (Lambda-cyhalothrin, malathion, novacron, cartap hydrochloride)	400 (19–148 mg/kg)			[4]
QuEChERS	GC-MS/MS	124		LOD:0.1–7.0 μg/kg LOQ: 0.4–26.3 μg/kg	70%–122.7%	[1]
Modified QuEChERS	LC-MS/MS	41	60 (11 domestic samples and 1 imported sample contaminated)	LOD: 0.008 μg/g LOQ: 0.025 μg/g	71%–119%	[13]
QuEChERS	LC-MS/MS	18 herbicides (12 quant.)	8 (all negative)	LOQ: 0.015–0.165 μg/g	92%–103%	[14]
QuEChERS	LC-MS/MS	20		LOQ 5–20 μg/kg	81%–123%	[15]

**Table 2:** Gradient elution program for the determination of pesticide residues in rice by high-performance liquid chromatography-tandem mass spectrometry (HPLC-MS/MS).

Time	Mobile Phase [A]	Mobile Phase [B]
**0**	95	5
**0.5**	95	5
**8**	10	90
**13**	10	90
**15**	95	5
**18**	95	5

**Table 3:** Results of the validation of the HPLC-MS/MS method to determine 155 pesticides in rice: determination coefficient (*r*^2^) in solvent and matrix-matched curves, recovery, and repeatability (RSD_r_) and precision (RSD_R_) at three different spiking levels, expanded uncertainty (U) and matrix effect (ME).

Pesticide	Linear Range Solvent (µg/L)	*r* ^2solvent^	Linear Range Matrix (µg/L)	r^2matrix^	Spiked Level 0.005 mg/kg		Spiked Level 0.01 mg/kg		Spiked Level 0.05 mg/kg		Precision RSD_R_%	Recovery%	U %	ME %
					Rec. %	RSD_r_ %*n* = 5	Rec. %	RSD_r_ %*n* = 5	Rec. %	RSD_r_ %*n* = 5				
**Acetamiprid**	5–60	0.9942	5–60	0.9891	102	10.7	104	6.8	106	8.9	8.8	104	16	93
**Azoxystrobin**	5–50	0.9822	5–50	0.9915	89	8.3	96	11.4	93	10.4	10.1	93	26	120
**Bixafen**	5–50	0.9959	5–50	0.9957	100	8.7	100	9.0	90	13.3	10.3	97	17	110
**Boscalid**	5–50	0.9991	5–60	0.9897	103	13.9	103	8.2	101	15.2	12.4	103	18	94
**Bupirimate**	5–50	0.9990	5–50	0.9943	99	7.0	100	8.3	96	16.8	10.7	98	17	101
**Buprofezin**	5–50	0.9967	5–50	0.9912	99	9.6	96	6.8	88	7.9	8.1	94	17	110
**Cadusafos**	5–50	0.9962	5–50	0.9977	84	5.2	84	10.9	82	6.8	7.6	83	29	111
**Carbaryl**	5–60	0.9983	5–60	0.9966	103	5.8	95	9.6	93	7.4	7.6	97	18	116
**Carbendazim**	5–60	0.9990	5–60	0.9978	86	12.6	85	12.8	93	5.4	10.3	88	28	120
**Carbofuran**	5–60	0.9999	5–60	0.9989	107	5.0	101	9.1	97	8.7	7.6	102	17	110
**Carbofuran-3-hydroxi**	5–60	0.9974	5–60	0.9979	99	6.2	94	9.8	96	7.2	7.7	96	16	105
**Carboxin**	5–60	0.9921	5–60	0.9935	93	11.6	97	9.5	88	12.5	11.2	93	20	105
**Chlorantraniliprole**	5–60	0.9917	5–60	0.9928	100	11.2	106	8.1	106	7.1	8.8	104	18	109
**Chlorfenvinphos**	5–60	0.9994	5–60	0.9997	105	3.0	99	7.2	94	5.1	5.2	100	14	111
**Chlorpirifos**	5–60	0.9934	5–60	0.9974	105	9.5	104	9.3	98	10.4	9.7	102	19	94
**Chlorpyrifos-methyl**	5–60	0.9950	5–60	0.9997	104	3.1	96	6.7	90	4.4	4.8	97	15	101
**Clofentezine**	5–60	0.9995	5–60	0.9965	96	10.3	93	8.3	89	5.5	8.0	93	24	79
**Clothianidin**	5–70	0.9966	5–60	0.9965	96	8.6	90	6.9	90	11.0	8.8	92	22	93
**Coumaphos**	5–60	0.9988	5–60	0.9970	94	7.3	95	6.6	92	7.1	7.0	94	19	85
**Cymoxanil**	5–50	0.9946	5–60	0.9941	106	8.6	104	6.4	101	6.8	7.2	103	14	104
**Cyproconazol**	5–60	0.9930	5–60	0.9981	111	5.1	108	7.5	103	10.6	7.7	108	20	105
**Cyprodinil**	5–50	0.9935	5–50	0.9968	97	7.0	88	8.9	88	5.7	7.2	91	28	105
**Demeton-S-methylsulfone**	5–60	0.9987	5–60	0.9924	99	5.2	100	9.6	101	4.5	6.4	100	14	104
**Desmethyl-pirimicarb**	5–60	0.9865	5–50	0.9863	91	6.5	88	6.9	86	6.7	6.7	88	27	95
**Diazinon**	5–60	0.9960	5–60	0.9972	102	8.0	102	4.2	104	5.5	5.9	103	12	92
**Dichlorvos**	5–60	0.9978	5–60	0.9978	102	6.7	96	9.9	91	6.3	7.6	97	18	108
**Dicrotophos**	5–60	0.9964	5–60	0.9997	105	3.0	99	5.6	91	6.1	4.9	98	15	116
**Diethofencarb**	5–50	0.9928	5–60	0.9962	97	9.6	97	9.4	96	14.1	11.1	97	18	98
**Difenoconazole**	5–50	0.9976	5–50	0.9980	107	4.2	106	5.0	96	10.8	6.7	103	16	112
**Diflubenzuron**	5–60	0.9980	5–60	0.9991	98	6.2	94	6.5	91	4.8	5.8	94	20	105
**Dimethoate**	5–60	0.9957	5–60	0.9995	106	4.9	96	8.3	90	6.9	6.7	97	19	112
**Dimethomorph**	5–50	0.9973	5–60	0.9885	95	7.3	97	12.9	99	6.4	8.9	97	19	103
**Diniconazole**	5–50	0.9964	5–50	0.9900	97	8.1	100	8.9	100	10.1	9.1	99	18	96
**EPN**	5–60	0.9967	5–60	0.9988	98	3.8	88	10.4	85	7.0	7.1	90	26	89
**Epoxiconazole**	5–50	0.9877	5–50	0.9959	112	2.6	108	5.2	104	8.4	5.4	108	17	112
**Ethiofencarb**	5–60	0.9980	5–60	0.9989	105	13.9	101	8.3	89	14.9	12.4	98	20	101
**Ethion**	5–60	0.9947	5–60	0.9941	99	10.8	101	9.2	100	6.5	8.8	100	16	100
**Ethirimol**	5–50	0.9924	5–60	0.9874	87	9.9	86	10.1	94	16.1	12.0	89	26	107
**Ethoprophos**	5–60	0.9988	5–60	0.9990	96	5.4	98	9.0	95	3.8	6.1	97	13	113
**Etrinphos**	5–60	0.9968	5–60	0.9979	82	6.5	109	5.9	98	5.2	5.9	96	20	111
**Fenamidone**	5–50	0.9837	5–60	0.9934	102	3.2	105	5.8	103	11.3	6.8	104	14	108
**Fenamiphos**	5–60	0.9989	5–60	0.9984	99	4.3	99	8.5	95	6.1	6.3	97	13	106
**Fenamiphos sulfone**	5–60	0.9987	5–60	0.9993	111	4.7	100	7.5	95	5.9	6.1	102	17	108
**Fenamiphos sulfoxide**	5–60	0.9979	5–60	0.9963	99	3.3	98	8.0	99	6.8	6.0	99	13	104
**Fenarimol**	5–50	0.9966	5–50	0.9982	102	7.6	99	9.6	89	10.4	9.2	97	17	109
**Fenezaquin**	5–60	0.9940	5–50	0.9817	94	9.0	78	5.8	n.v.	n.v.	7.4	86	20	109
**Fenhexamid**	5–50	0.9988	5–50	0.9988	96	16.5	94	10	87	13.5	13.2	92	25	111
**Fenitrothion**	5–50	0.9907	5–50	0.9933	100	10.4	97	12.8	99	10.3	11.2	99	22	93
**Fenoxycarb**	5–60	0.9988	5–60	0.9983	99	6.0	96	8.7	94	3.8	6.1	97	14	104
**Fenpropathrin**	5–50	0.9976	5–50	0.9824	98	10.3	83	11.4	76	7.0	9.6	86	36	99
**Fenpropidin**	5–70	0.9973	5–60	0.9964	110	5.6	99	7.7	95	9.2	7.5	101	18	91
**Fenpropimorph**	5–60	0.9959	5–60	0.9893	108	5.7	107	4.2	100	9.9	6.6	105	18	98
**Fenthion**	5–60	0.9986	5–60	0.9975	85	8.3	92	4.9	98	4.1	5.8	91	23	88
**Fenthion oxon**	5–60	0.9996	5–50	0.9994	94	5.6	94	7.8	93	6.2	6.5	94	19	104
**Fenthion oxon sulfone**	5–60	0.9981	5–60	0.9996	108	3.5	95	7.9	89	5.5	5.6	97	19	104
**Fenthion oxon sulfoxide**	5–60	0.9955	5–60	0.9993	102	3.7	93	9.4	87	6.8	6.6	94	19	114
**Fenthion sulfoxide**	5–60	0.9995	5–60	0.9995	105	3.3	98	7.8	92	4.9	5.3	98	16	108
**Fenthion-sulfone**	5–60	0.9990	5–60	0.9993	110	1.6	97	8.7	90	5.0	5.1	99	21	109
**Fipronil**	5–50	0.9912	5–50	0.9908	75	6.8	94	8.0	81	9.7	8.2	83	23	123
**Fludioxonil**	5–60	0.9961	5–50	0.9979	98	15.2	106	8.7	99	7.7	10.5	101	24	105
**Flufenoxuron**	5–70	0.9978	5–50	0.9910	100	9.5	101	14.8	95	18.6	14.3	99	20	57
**Fluopyram**	5–50	0.9817	5–50	0.9965	105	4.6	99	9.5	96	9.9	8.0	100	15	110
**Fluquinconazole**	5–50	0.9957	5–50	0.9915	109	1.2	100	8.1	91	11.5	6.9	100	21	106
**Flusilazole**	5–50	0.9978	5–60	0.9971	98	10.3	104	11.9	103	10.1	10.8	101	22	108
**Flutriafol**	5–50	0.9913	5–60	0.9888	112	4.1	111	4.5	112	6.0	4.9	111	19	95
**Fonofos**	5–60	0.9955	5–60	0.9987	94	8.1	93	10.3	94	5.3	7.9	94	21	103
**Fosthiazate**	5–60	0.9994	5–60	0.9994	93	5.7	89	8.7	89	6.0	6.8	90	21	105
**Hexaconazole**	5–50	0.9975	5–60	0.9943	111	2.0	104	6.7	99	10.3	6.3	105	18	110
**Hexythiazox**	5–50	0.9933	5–60	0.9875	96	16.8	89	10.3	86	11.0	12.7	90	30	103
**Imazalil**	5–50	0.9931	5–60	0.9935	107	8.8	104	9.6	106	8.2	8.9	106	17	120
**Imidacloprid**	5–70	0.9962	5–50	0.9950	103	5.5	96	7.1	96	11.1	7.9	99	16	91
**Indoxacarb**	5–60	0.9908	5–60	0.9864	102	13.1	102	11.2	107	8.3	10.9	104	22	103
**Iprodione**	5–50	0.9933	5–50	0.9988	107	10.3	98	9.2	87	9.6	9.7	97	19	101
**Iprovalicarb**	5–60	0.9966	5–60	0.9880	84	13.2	82	9.2	92	9.6	10.7	86	34	93
**Isoprocarb**	5–60	0.9998	5–60	0.9990	99	6.7	94	10.0	91	7.0	8.0	95	13	111
**Isoprothiolane**	5–60	0.9882	5–60	0.9948	106	9.0	106	8.5	98	11.2	9.6	103	18	104
**Isoproturon**	5–60	0.9964	5–60	0.9952	87	7.8	93	5.0	99	5.3	6.0	93	24	96
**Kresoxim-methyl**	5–50	0.9943	5–50	0.9809	97	15.1	94	15.1	88	9.9	13.4	93	21	114
**Linuron**	5–50	0.9922	5–50	0.9917	97	8.4	92	7.3	94	4.0	6.6	94	21	107
**Lufenuron**	5–50	0.9964	5–60	0.9935	94	14.0	93	16.9	76	5.9	12.3	88	25	78
**Malaoxon**	5–60	0.9981	5–60	0.9977	101	6.2	97	7.7	95	6.2	6.7	98	14	109
**Malathion**	5–60	0.9996	5–60	0.9998	96	5.8	94	6.5	94	4.4	5.6	95	17	108
**Mandipropamid**	5–50	0.9941	5–50	0.9918	84	4.2	83	5.6	90	4.2	4.7	86	29	103
**Mepanipyrim**	5–50	0.9922	5–60	0.9976	79	5.0	82	5.2	88	7.9	6.0	83	33	105
**Metaflumizone**	5–50	0.9937	5–50	0.9807	95	17.9	111	4.5	97	10.1	10.8	101	27	50
**Metalaxyl**	5–60	0.9921	5–50	0.9939	75	4.0	81	4.4	80	4.8	4.4	78	37	108
**Metalaxyl-M**	5–60	0.9899	5–50	0.9939	98	9.9	106	6.3	99	9.9	8.7	101	18	110
**Metazachlor**	5–50	0.9905	5–60	0.9847	74	4.4	80	3.6	81	7.5	5.2	78	37	120
**Metconazole**	5–50	0.9898	5–50	0.9970	82	7.2	88	5.2	90	5.4	5.9	87	29	113
**Methacrifos**	5–50	0.9914	5–50	0.9937	83	10.3	93	12.4	91	6.1	9.6	89	27	100
**Methiocarb**	5–60	0.9993	5–60	0.9985	87	5.3	85	10.5	86	3.1	6.3	86	27	99
**Methiocarb sulfoxide**	5–60	0.9969	5–60	0.9964	91	6.4	75	2.2	74	5.0	4.6	80	43	112
**Methomyl**	5–50	0.9862	5–60	0.9866	107	8.0	105	6.9	97	7.1	7.3	103	16	135
**Methoxyfenozide**	5–50	0.9957	5–60	0.9994	88	17.7	90	9.8	90	14.0	13.8	90	24	111
**Metobromuron**	5–60	0.9993	5–60	0.9994	103	6.4	93	7.1	94	5.1	6.2	97	16	103
**Metribuzin**	5–50	0.9901	5–60	0.9952	77	6.8	75	4.1	81	8.8	6.5	78	39	113
**Mevinfos**	5–60	0.9912	5–60	0.9917	108	4.8	103	6.4	104	7.7	6.3	105	18	101
**Monocrotophos**	5–50	0.9905	5–60	0.9851	87	9.7	93	8.3	88	5.9	8.0	89	28	106
**Myclobutanil**	5–50	0.9948	5–50	0.9967	97	7.6	97	4.1	101	8.0	6.6	98	12	102
**N,N-dimethyl-N’-p-tolysulphamide**	5–60	0.9804	5–60	0.9968	86	10.7	91	7.6	93	6.2	8.1	90		85
**Nitenpyram**	5–50	0.9962	5–50	0.9799	99	10.9	87	7.6	85	7.6	8.7	90	23	112
**Omethoate**	5–60	0.9989	5–60	0.9948	86	12.3	86	9.3	75	4.9	8.8	83	29	114
**Oxadixyl**	5–60	0.9951	5–60	0.9880	107	6.2	105	4.6	106	7.9	6.2	106	16	107
**Oxidemeton methyl**	5–60	0.9978	5–60	0.9991	99	4.0	88	5.9	86	4.2	4.7	91	27	121
**Paclobutrazol**	5–50	0.9979	5–50	0.9988	86	5.1	93	6.6	97	5.5	5.7	92	25	111
**Paraoxon-ethyl**	5–60	0.9992	5–60	0.9992	97	6.6	94	8.8	96	5.0	6.8	96	14	110
**Paraoxon-methyl**	5–60	0.9962	5–60	0.9987	112	3.1	98	9.8	90	6.2	6.4	100	21	110
**Parathion**	5–60	0.9892	5–60	0.9953	97	4.2	93	8.2	88	3.5	5.3	93	21	102
**Parathion-methyl**	5–60	0.9856	5–60	0.9831	80	7.7	84	8.1	100	2.8	6.2	88	29	67
**Penconazole**	5–50	0.9920	5–50	0.9985	90	5.9	90	6.6	90	5.3	5.9	90	23	113
**Pencycuron**	5–60	0.9961	5–60	0.9988	103	2.8	102	3.6	104	3.2	3.2	103	10	100
**Pendimethalin**	5–60	0.9945	5–60	0.9889	104	5.2	92	14.4	85	15.1	11.6	94	25	87
**Phenthoate**	5–60	0.9990	5–60	0.9985	109	4.2	101	7.6	95	5.2	5.7	101	16	104
**Phosalone**	5–60	0.9934	5–60	0.9990	93	7.2	86	9.7	84	7.3	8.0	88	26	102
**Phosmet**	5–50	0.9977	5–60	0.9925	88	16.2	88	15.2	81	10.0	13.8	86	35	97
**Phosphamidon**	5–60	0.9860	5–60	0.9926	97	9.5	93	8.5	90	5.8	7.9	93	23	105
**Phoxim**	5–50	0.9923	5–50	0.9958	85	10.3	92	10.8	94	6.0	9.0	90	28	101
**Pirimicarb**	5–60	0.9907	5–60	0.9865	94	11.6	104	7.6	114	5.0	8.1	104	20	40
**Pirimiphos-ethyl**	5–50	0.9853	5–60	0.9970	76	6.0	77	4.4	80	7.7	6.0	77	34	119
**Pirimiphos-methyl**	5–50	0.9956	5–60	0.9981	80	8.7	85	5.2	80	7.2	7.0	82	30	109
**Prochloraz**	5–50	0.9956	5–60	0.9948	89	5.6	87	7.2	90	4.5	5.8	89	27	114
**Profenofos**	5–60	0.9944	5–60	0.9916	87	14.1	82	6.4	76	9.0	9.8	81	36	109
**Propiconazol**	5–50	0.9934	5–50	0.9978	103	5.7	94	5.4	96	5.0	5.4	98	14	111
**Propoxur**	5–60	0.9987	5–60	0.9987	113	4.7	101	8.5	93	6.7	6.7	102	21	108
**Propyzamide**	5–50	0.9959	5–50	0.9957	81	8.8	86	3.9	90	8.7	7.1	86	28	104
**Prothioconazole-desthio**	5–50	0.9935	5–50	0.9944	94	3.1	95	5.8	105	6.2	5.0	98	16	109
**Pyraclostrobin**	5–60	0.9950	5–50	0.9982	101	11.1	99	8.9	97	5.8	8.6	99	20	106
**Pyrazophos**	5–60	0.9878	5–60	0.9985	98	6.2	103	8.6	99	3.5	6.1	100	14	113
**Pyridaben**	5–50	0.9947	5–50	0.9733	116	3.2	98	15.3	82	7.1	8.5	99	28	82
**Pyrimethanil**	5–60	0.9945	5–60	0.9847	109	7.9	109	5.7	104	9.0	7.5	107	21	96
**Pyriproxyfen**	5–50	0.9913	5–50	0.9994	75	3.9	81	8.7	79	6.5	6.4	78	38	115
**Quinoxyfen**	5–50	0.9912	5–60	0.9995	84	9.1	79	7.6	78	8.5	8.4	80	36	142
**Rotenone**	5–50	0.9892	5–50	0.9963	80	5.4	84	8.3	89	9.6	7.8	85	32	107
**Spinosad A**	5–60	0.9899	5–50	0.9988	107	6.2	101	10.5	98	8.9	8.5	102	17	97
**Spinosad D**	5–60	0.9869	5–60	0.9973	105	8.7	99	15.7	94	2.8	9.1	99	20	92
**Spiroxamine**	5–60	0.9926	5–60	0.9868	113	2.8	108	4.8	93	10.3	6.0	105	20	103
**Tebuconazol**	5–50	0.9954	5–50	0.9963	75	6.6	86	6.1	89	5.3	6.0	83	30	111
**Tebufenpyrad**	5–50	0.9868	5–60	0.9970	85	6.8	88	9.3	89	8.1	8.1	87	28	116
**Teflubenzuron**	5–60	0.9966	5–50	0.9967	97	17.7	94	17.4	n.v.	n.v.	17.5	96	27	38
**Terbuthylazine**	5–50	0.9918	5–60	0.9986	73	3.1	77	5.6	82	7.5	5.4	77	11	114
**Tetraconazole**	5–50	0.9919	5–50	0.9939	88	5.0	85	6.1	92	7.3	6.1	88	27	113
**Tetramethrin**	5–50	0.9863	5–50	0.9866	83	6.8	85	10.3	99	11.9	9.6	89	27	90
**Thiabendazole**	5–60	0.9961	5–60	0.9994	113	5.5	103	3.9	93	5.4	5.0	103	19	125
**Thiacloprid**	5–60	0.9851	5–60	0.9926	76	4.9	77	4.7	82	6.2	5.3	78	32	109
**Thiamethoxam**	5–50	0.9954	5–60	0.9872	108	8.0	103	9.4	93	6.4	7.9	101	18	106
**Thiodicarb**	5–60	0.9983	5–60	0.9917	103	12.5	93	10.2	92	13.3	12.0	96	20	95
**Thiophanate-methyl**	5–60	0.9981	5–60	0.9874	103	7.0	99	6.3	101	9.5	7.6	101	17	132
**Tolclofos-methyl**	5–50	0.9949	5–60	0.9953	84	10.3	85	8.8	85	10.1	9.7	84	13	100
**Triadimefon**	5–50	0.9964	5–50	0.9965	77	4.8	87	5.1	105	4.3	4.7	90	33	104
**Triadimenol**	5–50	0.9814	5–50	0.9976	90	5.5	90	8.2	99	5.7	6.5	93	24	114
**Triazophos**	5–50	0.9930	5–50	0.9978	81	6.0	90	7.5	94	5.7	6.4	88	28	105
**Tricyclazole**	5–60	0.9969	5–60	0.9691	105	1.8	107	4.0	104	7.1	4.3	108	14	95
**Trifloxystrobin**	5–50	0.9894	5–60	0.9932	78	6.3	79	6.1	77	7.5	6.6	78	35	117
**Triflumuron**	5–60	0.9907	5–60	0.9996	88	9.2	84	9.2	86	5.5	8.0	86	28	103
**Zoxamide**	5–50	0.9978	5–60	0.9973	78	3.5	82	5.1	81	8.2	5.6	80	17	104

n.v.—not validated.

## Supplementary Materials

The following are available online at https://www.mdpi.com/2304-8158/9/1/18/s1, Table S1: Parameters for determination of pesticides residues in rice by HPLC-MS/MS in ESI+ mode. Transition 1: Quantification transition; Transition 2: Confirmation transition, Table S2: Parameters for determination of pesticides residues in rice by HPLC-MS/MS in ESI- mode. Transition 1: Quantification transition; Transition 2: Confirmation transition.
