# Supplementary material for: Modified QuEChERS Extraction and HPLC-MS/MS for Simultaneous Determination of 155 Pesticide Residues in Rice (Oryza sativa L.)
Source: Foods. 2019 Dec 24;9(1):18. doi: 10.3390/foods9010018 (PMC7022397; doi:10.3390/foods9010018)
Supplement: Supplementary file 1 [file foods-09-00018-s001.pdf]

foods

MDPI

*Supplementary material*

**Table S1.** Parameters for determination of pesticides residues in rice by HPLC-MS/MS in ESI+ mode. Transition 1: Quantification transition; Transition 2: Confirmation transition.

| Pesticide | Functional class | Transitions | Precursor ion (m/z ±0.5) | Product ion (m/z ±0.5) | DP (V) | CE (V) | CXP (V) |
|---|---|---|---|---|---|---|---|
| **TPP** | | | 327 | 152 | 106 | 47 | 10 |
| Acetamiprid | Insecticide, acaricide | Transition 1 | 223 | 126 | 74 | 27 | 4 |
| | | Transition 2 | 225 | 128 | 61 | 29 | 8 |
| Azoxystrobin | Fungicide | 1 | 404 | 372 | 46 | 21 | 10 |
| | | 2 | 404 | 344 | 46 | 35 | 20 |
| Bixafen | Fungicide | 1 | 414 | 394 | 76 | 21 | 24 |
| | | 2 | 416 | 396 | 81 | 23 | 16 |
| Boscalid | Fungicide | 1 | 343 | 307 | 76 | 29 | 18 |
| | | 2 | 343 | 140 | 116 | 27 | 6 |
| Bupirimate | Fungicide | 1 | 317 | 272 | 61 | 27 | 22 |
| | | 2 | 317 | 108 | 91 | 41 | 6 |
| Buprofezin | Insecticide | 1 | 306 | 201 | 51 | 17 | 14 |
| | | 2 | 306 | 116 | 56 | 23 | 20 |
| Cadusafos | Insecticide | 1 | 271 | 159 | 86 | 19 | 6.5 |
| | | 2 | 271 | 131 | 71 | 31 | 6.5 |
| Carbaryl | Insecticide, plant growth regulator | 1 | 202 | 145 | 86 | 17 | 16 |
| | | 2 | 202 | 127 | 86 | 28 | 14 |
| Carbendazim | Fungicide | 1 | 192 | 160 | 51 | 25 | 10 |
| | | 2 | 192 | 105 | 61 | 51 | 18 |

| Pesticide | Functional class | Transitions | Precursor ion (m/z ±0.5) | Product ion (m/z ±0.5) | DP (V) | CE (V) | CXP (V) |
|---|---|---|---|---|---|---|---|
| Carbofuran | Insecticide | 1 | 222 | 165 | 56 | 15 | 22 |
| | | 2 | 222 | 123 | 56 | 25 | 14 |
| Carbofuran-3-hydroxi | Insecticide | 1 | 238 | 181 | 82 | 17 | 10 |
| | | 2 | 238 | 163 | 82 | 21 | 10 |
| Carboxin | Fungicide | 1 | 236 | 143 | 46 | 23 | 8 |
| | | 2 | 236 | 87 | 46 | 35 | 14 |
| Cymoxanil | Fungicide | 1 | 199 | 128 | 46 | 13 | 8 |
| | | 2 | 199 | 83 | 46 | 33 | 14 |
| Cyproconazol | Fungicide | 1 | 292 | 70 | 71 | 35 | 4 |
| | | 2 | 294 | 125 | 16 | 35 | 14 |
| Cyprodinil | Fungicide | 1 | 226 | 91 | 81 | 49 | 16 |
| | | 2 | 226 | 93 | 81 | 47 | 16 |
| Clofentezine | Insecticide, acaricide | 1 | 303 | 138 | 56 | 21 | 20 |
| | | 2 | 303 | 102 | 56 | 59 | 18 |
| Chlorfenvinphos | Insecticide | 1 | 359 | 99 | 75 | 41 | 6 |
| | | 2 | 359 | 155 | 75 | 19 | 8 |
| Chlorantraniliprole | Insecticide | 1 | 484 | 286 | 61 | 21 | 16 |
| | | 2 | 484 | 453 | 61 | 23 | 6 |
| Chlorpyrifos-methyl | Insecticide | 1 | 324 | 125 | 66 | 25 | 8 |
| | | 2 | 322 | 125 | 66 | 29 | 6 |
| Chlorpirifos | Insecticide | 1 | 350 | 97 | 21 | 47 | 6 |
| | | 2 | 350 | 198 | 80 | 25 | 6 |
| Clothianidin | Insecticide | 1 | 250 | 169 | 76 | 19 | 13 |
| | | 2 | 250 | 132 | 94 | 19 | 13 |

| Pesticide | Functional class | Transitions | Precursor ion (m/z ±0.5) | Product ion (m/z ±0.5) | DP (V) | CE (V) | CXP (V) |
|---|---|---|---|---|---|---|---|
| Coumaphos | Insecticide | 1 | 363 | 227 | 84 | 37 | 12 |
| | | 2 | 363 | 307 | 84 | 23 | 7 |
| Demeton-S-methylsulfone | Insecticide | 1 | 263 | 109 | 76 | 37 | 6 |
| | | 2 | 263 | 169 | 76 | 16 | 6 |
| Desmethyl-pirimicarb | Insecticide | 1 | 225 | 72 | 56 | 27 | 7 |
| | | 2 | 225 | 168 | 61 | 21 | 8 |
| Diazinon | Insecticide, acaricide | 1 | 305 | 100 | 76 | 49 | 4 |
| | | 2 | 305 | 97 | 76 | 95 | 4 |
| Dichlorvos | Insecticide, acaricide | 1 | 221 | 109 | 76 | 25 | 4 |
| | | 2 | 223 | 109 | 66 | 23 | 18 |
| Dicrotophos | Insecticide | 1 | 238 | 112 | 70 | 17 | 6 |
| | | 2 | 238 | 193 | 70 | 15 | 8 |
| Diethofencarb | Fungicide | 1 | 268 | 124 | 61 | 43 | 6 |
| | | 2 | 268 | 226 | 61 | 15 | 12 |
| Difenoconazole | Fungicide | 1 | 406 | 251 | 81 | 37 | 4 |
| | | 2 | 406 | 337 | 41 | 23 | 13 |
| Diflubenzuron | Insecticide | 1 | 311 | 158 | 81 | 19 | 18 |
| | | 2 | 311 | 141 | 81 | 43 | 20 |
| Dimethoate | Insecticide, acaricide | 1 | 230 | 199 | 61 | 13 | 8 |
| | | 2 | 230 | 125 | 61 | 29 | 6 |
| Dimethomorph | Fungicide | 1 | 388 | 301 | 51 | 45 | 10 |
| | | 2 | 388 | 165 | 51 | 29 | 18 |
| DMST | Fungicide | 1 | 215 | 106 | 100 | 10 | 13 |
| | | 2 | 215 | 151 | 100 | 5 | 13 |

| Pesticide | Functional class | Transitions | Precursor ion (m/z ±0.5) | Product ion (m/z ±0.5) | DP (V) | CE (V) | CXP (V) |
|---|---|---|---|---|---|---|---|
| Diniconazole | Fungicide | 1 | 326 | 159 | 56 | 39 | 13 |
| | | 2 | 326 | 70 | 56 | 45 | 13 |
| EPN | Insecticide, acaricide | 1 | 324 | 296 | 16 | 19 | 6 |
| | | 2 | 324 | 157 | 16 | 29 | 6 |
| Epoxiconazole | Fungicide | 1 | 330 | 101 | 36 | 63 | 13 |
| | | 2 | 330 | 121 | 36 | 27 | 13 |
| Spiroxamine | Fungicide | 1 | 298 | 144 | 41 | 27 | 10 |
| | | 2 | 298 | 100 | 41 | 41 | 6 |
| Ethion | Insecticide | 1 | 385.1 | 97 | 61 | 53 | 4 |
| | | 2 | 385 | 199 | 61 | 13 | 4 |
| Ethiofencarb | Insecticide | 1 | 226 | 107 | 51 | 23 | 18 |
| | | 2 | 226 | 164 | 51 | 13 | 10 |
| Ethirimol | Fungicide | 1 | 210 | 140 | 71 | 31 | 22 |
| | | 2 | 210 | 98 | 66 | 37 | 18 |
| Ethoprophos | Insecticide, nematicide | 1 | 243 | 97 | 71 | 41 | 4 |
| | | 2 | 243 | 131 | 66 | 29 | 8 |
| Etrinphos | Insecticide | 1 | 293 | 125 | 64 | 33 | 8 |
| | | 2 | 293 | 265 | 61 | 21 | 10 |
| Fenamidone | Fungicide | 1 | 312 | 65 | 81 | 71 | 4 |
| | | 2 | 312 | 236 | 41 | 19 | 4 |
| Fenamiphos | Nematocide | 1 | 304 | 217 | 81 | 31 | 4 |
| | | 2 | 304 | 202 | 81 | 47 | 4 |
| Fenamiphos sulfone | Nematocide | 1 | 336 | 266 | 81 | 29 | 13 |
| | | 2 | 336 | 188 | 81 | 41 | 13 |

| Pesticide | Functional class | Transitions | Precursor ion (m/z ±0.5) | Product ion (m/z ±0.5) | DP (V) | CE (V) | CXP (V) |
|---|---|---|---|---|---|---|---|
| Fenamiphos sulfoxide | Nematocide | 1 | 320 | 233 | 86 | 35 | 12 |
| | | 2 | 320 | 108 | 86 | 59 | 6 |
| Fenarimol | Fungicide | 1 | 331 | 268 | 86 | 31 | 10 |
| | | 2 | 331 | 139 | 96 | 49 | 14 |
| Fenpropathrin | Acaricide, insecticide | 1 | 350 | 97 | 81 | 43 | 4 |
| | | 2 | 350 | 125 | 76 | 19 | 6 |
| Fenpropidin | Fungicide | 1 | 274 | 147 | 51 | 37 | 7 |
| | | 2 | 274 | 117 | 51 | 65 | 7 |
| Fenpropimorph | Fungicide | 1 | 304 | 147 | 74 | 39 | 7 |
| | | 2 | 304 | 117 | 11 | 71 | 6 |
| Fenezaquin | Insecticide | 1 | 307 | 161 | 51 | 31 | 13 |
| | | 2 | 307 | 147 | 51 | 25 | 13 |
| Fenhexamid | Fungicide | 1 | 302 | 55 | 96 | 59 | 4 |
| | | 2 | 302 | 97 | 91 | 35 | 6 |
| Fenitrothion | Insecticide | 1 | 278 | 125 | 71 | 31 | 6 |
| | | 2 | 278 | 109 | 71 | 25 | 6 |
| Fenoxycarb | Insecticide | 1 | 302 | 116 | 36 | 15 | 16 |
| | | 2 | 302 | 88 | 36 | 23 | 16 |
| Fenthion | Insecticide | 1 | 279 | 169 | 56 | 25 | 14 |
| | | 2 | 279 | 247 | 56 | 19 | 16 |
| Fenthion oxon | Insecticide | 1 | 263 | 231 | 66 | 23 | 14 |
| | | 2 | 263 | 216 | 76 | 33 | 14 |
| Fenthion oxon sulfone | Insecticide | 1 | 295 | 217 | 96 | 27 | 12 |
| | | 2 | 295 | 104 | 96 | 35 | 6 |

| Pesticide | Functional class | Transitions | Precursor ion (m/z ±0.5) | Product ion (m/z ±0.5) | DP (V) | CE (V) | CXP (V) |
|---|---|---|---|---|---|---|---|
| Fenthion oxon sulfoxide | Insecticide | 1 | 279 | 264 | 56 | 27 | 18 |
| | | 2 | 279 | 104 | 56 | 39 | 18 |
| Fenthion-sulfone | Insecticide | 1 | 311 | 125 | 76 | 28 | 8 |
| | | 2 | 311 | 279 | 76 | 27 | 16 |
| Fenthion sulfoxide | Insecticide | 1 | 295 | 280 | 76 | 25 | 16 |
| | | 2 | 295 | 109 | 76 | 45 | 18 |
| Phenthoate | Insecticide, acaricide | 1 | 321 | 79 | 36 | 51 | 6 |
| | | 2 | 321 | 247 | 16 | 13 | 10 |
| Flufenoxuron | Insecticide | 1 | 489 | 158 | 31 | 25 | 10 |
| | | 2 | 489 | 141 | 31 | 73 | 20 |
| Fluopyram | Fungicide | 1 | 397 | 145 | 81 | 79 | 8 |
| | | 2 | 397 | 173 | 81 | 43 | 10 |
| Fluquinconazole | Fungicide | 1 | 376 | 307 | 56 | 33 | 7 |
| | | 2 | 376 | 349 | 56 | 25 | 14 |
| Flusilazole | Fungicide | 1 | 316 | 247 | 76 | 27 | 14 |
| | | 2 | 316 | 165 | 76 | 37 | 12 |
| Flutriafol | Fungicide | 1 | 302 | 123 | 41 | 39 | 13 |
| | | 2 | 302 | 109 | 41 | 43 | 13 |
| Fonofos | Insecticide | 1 | 247 | 109 | 61 | 26 | 6 |
| | | 2 | 247 | 137 | 61 | 15 | 6 |
| Phosalone | Insecticide | 1 | 368 | 182 | 66 | 19 | 14 |
| | | 2 | 368 | 111 | 51 | 51 | 18 |
| Phosphamidon | Insecticide, acaricide | 1 | 300 | 127 | 66 | 27 | 6 |
| | | 2 | 300 | 227 | 66 | 19 | 14 |

| Pesticide | Functional class | Transitions | Precursor ion (m/z ±0.5) | Product ion (m/z ±0.5) | DP (V) | CE (V) | CXP (V) |
|---|---|---|---|---|---|---|---|
| Phosmet | Insecticide, acaricide | 1 | 318.1 | 160 | 56 | 21 | 12 |
| | | 2 | 318.1 | 77 | 56 | 69 | 13 |
| Fosthiazate | Insecticide, nematicide | 1 | 284 | 104 | 11 | 27 | 6 |
| | | 2 | 284 | 228 | 11 | 15 | 8 |
| Phoxim | Insecticide | 1 | 299 | 77 | 56 | 47 | 12 |
| | | 2 | 299 | 129 | 56 | 17 | 12 |
| Hexaconazole | Fungicide | 1 | 316 | 70 | 81 | 23 | 14 |
| | | 2 | 314 | 159 | 71 | 39 | 4 |
| Hexythiazox | Acaricide | 1 | 353 | 228 | 66 | 21 | 4 |
| | | 2 | 353 | 168 | 66 | 37 | 14 |
| Imazalil | Fungicide | 1 | 297 | 159 | 56 | 31 | 14 |
| | | 2 | 299 | 161 | 46 | 29 | 10 |
| Imidacloprid | Insecticide | 1 | 256 | 209 | 89 | 23 | 4 |
| | | 2 | 256 | 175 | 89 | 25 | 4 |
| Indoxacarb | Insecticide | 1 | 528 | 150 | 26 | 31 | 18 |
| | | 2 | 528 | 203 | 96 | 51 | 7 |
| Iprodione | Fungicide | 1 | 330 | 245 | 61 | 21 | 14 |
| | | 2 | 332 | 247 | 61 | 21 | 14 |
| Iprovalicarb | Fungicide | 1 | 321 | 119 | 56 | 27 | 8 |
| | | 2 | 321 | 186 | 56 | 17 | 16 |
| Isoprocarb | Insecticide | 1 | 194 | 95 | 65 | 19 | 14 |
| | | 2 | 194 | 152 | 65 | 11 | 20 |
| Isoprothiolane | Fungicide | 1 | 291 | 231 | 51 | 17 | 14 |
| | | 2 | 291 | 189 | 51 | 31 | 12 |

| Pesticide | Functional class | Transitions | Precursor ion (m/z ±0.5) | Product ion (m/z ±0.5) | DP (V) | CE (V) | CXP (V) |
|---|---|---|---|---|---|---|---|
| Isoproturon | Herbicide | 1 | 207 | 72 | 61 | 35 | 12 |
| | | 2 | 207 | 165 | 61 | 21 | 10 |
| Kresoxim-methyl | Fungicide | 1 | 314 | 267 | 75 | 5 | 13 |
| | | 2 | 314 | 222 | 75 | 9 | 13 |
| Linuron | Herbicide | 1 | 249 | 160 | 61 | 25 | 18 |
| | | 2 | 249 | 182 | 61 | 21 | 22 |
| Lufenuron | Insecticide | 1 | 511 | 158 | 79 | 27 | 4 |
| | | 2 | 511 | 141 | 79 | 67 | 4 |
| Malaoxon | Insecticide | 1 | 315 | 99 | 71 | 31 | 4 |
| | | 2 | 315 | 127 | 71 | 17 | 6 |
| Malathion | Insecticide | 1 | 331 | 127 | 64 | 17 | 4 |
| | | 2 | 331 | 285 | 64 | 13 | 4 |
| Mandipropamid | Fungicide | 1 | 412 | 328 | 76 | 21 | 16 |
| | | 2 | 412 | 125 | 76 | 55 | 8 |
| Mepanipyrim | Bactericide, fungicide | 1 | 224 | 106 | 66 | 35 | 7 |
| | | 2 | 224 | 77 | 66 | 49 | 7 |
| Methacrifos | Insecticide, acaricide | 1 | 241 | 209 | 71 | 13 | 6 |
| | | 2 | 241 | 125 | 61 | 27 | 6 |
| Metaflumizone | Insecticide | 1 | 507 | 178 | 86 | 35 | 10 |
| | | 2 | 507 | 89 | 86 | 123 | 4 |
| Metalaxyl | Fungicide | 1 | 280 | 192 | 61 | 25 | 10 |
| | | 2 | 280 | 220 | 61 | 21 | 12 |
| Metalaxyl-M | Fungicide | 1 | 280 | 220 | 46 | 19 | 13 |
| | | 2 | 280 | 160 | 46 | 31 | 13 |

| Pesticide | Functional class | Transitions | Precursor ion (m/z ±0.5) | Product ion (m/z ±0.5) | DP (V) | CE (V) | CXP (V) |
|---|---|---|---|---|---|---|---|
| Metazachlor | Herbicide | 1 | 278 | 210 | 41 | 15 | 4 |
| | | 2 | 278 | 134 | 41 | 29 | 8 |
| Metconazole | Fungicide | 1 | 320 | 70 | 76 | 45 | 7 |
| | | 2 | 320 | 125 | 81 | 49 | 6 |
| Methiocarb | Insecticide | 1 | 226 | 169 | 101 | 13 | 20 |
| | | 2 | 226 | 121 | 101 | 25 | 18 |
| Methiocarb sulfoxide | Insecticide | 1 | 242 | 185 | 56 | 19 | 10 |
| | | 2 | 242 | 122 | 56 | 41 | 10 |
| Metobromuron | Herbicide | 1 | 261 | 172 | 76 | 25 | 6.5 |
| | | 2 | 261 | 148 | 76 | 21 | 4 |
| Methomyl | Insecticide, acaricide | 1 | 163 | 88 | 46 | 13 | 4 |
| | | 2 | 163 | 106 | 46 | 15 | 18 |
| Metribuzin | Herbicide | 1 | 215 | 187 | 31 | 25 | 13 |
| | | 2 | 215 | 84.1 | 31 | 29 | 13 |
| Mevinfos | Insecticide, acaricide | 1 | 225 | 127 | 69 | 19 | 4 |
| | | 2 | 225 | 193 | 69 | 13 | 4 |
| Myclobutanil | Fungicide | 1 | 289 | 70 | 61 | 37 | 12 |
| | | 2 | 289 | 125 | 61 | 49 | 8 |
| Monocrotophos | Insecticide, acaricide | 1 | 224 | 127 | 86 | 21 | 6 |
| | | 2 | 224 | 193 | 86 | 13 | 14 |
| Nitenpyram | Insecticide | 1 | 271 | 225 | 79 | 15 | 4 |
| | | 2 | 271 | 99 | 79 | 21 | 4 |
| Omethoate | Insecticide, acaricide | 1 | 214 | 125 | 51 | 29 | 8 |
| | | 2 | 214 | 109 | 51 | 35 | 8 |

| Pesticide | Functional class | Transitions | Precursor ion (m/z ±0.5) | Product ion (m/z ±0.5) | DP (V) | CE (V) | CXP (V) |
|---|---|---|---|---|---|---|---|
| Oxadixyl | Fungicide | 1 | 279 | 219 | 66 | 15 | 4 |
| | | 2 | 279 | 132 | 66 | 41 | 4 |
| Oxidemeton methyl | Insecticide, acaricide | 1 | 247 | 169 | 71 | 19 | 6 |
| | | 2 | 247 | 109 | 8 | 40 | 7 |
| Paclobutrazol | Regulator of systemic plant growth | 1 | 294 | 70 | 71 | 39 | 4 |
| | | 2 | 294 | 89 | 71 | 81 | 4 |
| Paraoxon-ethyl | Insecticide | 1 | 276 | 220 | 61 | 19 | 6 |
| | | 2 | 276 | 248 | 61 | 15 | 16 |
| Paraoxon-methyl | Insecticide | 1 | 248 | 202 | 96 | 25 | 7 |
| | | 2 | 248 | 90 | 66 | 37 | 6 |
| Parathion | Insecticide | 1 | 292 | 236 | 81 | 21 | 6.5 |
| | | 2 | 292 | 264 | 81 | 15 | 13 |
| Parathion-methyl | Insecticide | 1 | 264 | 125 | 76 | 25 | 7 |
| | | 2 | 264 | 109 | 120 | 21 | 13 |
| Pencycuron | Fungicide | 1 | 329 | 125 | 71 | 33 | 8 |
| | | 2 | 329 | 218 | 71 | 23 | 14 |
| Penconazole | Fungicide | 1 | 284 | 70 | 61 | 49 | 12 |
| | | 2 | 284 | 159 | 61 | 47 | 14 |
| Pendimethalin | Herbicide | 1 | 282 | 212 | 41 | 17 | 12 |
| | | 2 | 282 | 194 | 41 | 27 | 12 |
| Pyraclostrobin | Fungicide | 1 | 388 | 194 | 51 | 19 | 16 |
| | | 2 | 388 | 163 | 51 | 35 | 14 |
| Pyrazophos | Fungicide | 1 | 374 | 222 | 81 | 29 | 6 |
| | | 2 | 374 | 194 | 11 | 43 | 7 |

| Pesticide | Functional class | Transitions | Precursor ion (m/z ±0.5) | Product ion (m/z ±0.5) | DP (V) | CE (V) | CXP (V) |
|---|---|---|---|---|---|---|---|
| Pyridaben | Acaricide | 1 | 365 | 147 | 26 | 31 | 13 |
| | | 2 | 365 | 309 | 26 | 19 | 13 |
| Pyrimethanil | Fungicide | 1 | 200 | 107 | 71 | 35 | 8 |
| | | 2 | 200 | 82 | 71 | 37 | 14 |
| Pirimicarb | Insecticide | 1 | 239 | 72 | 51 | 37 | 12 |
| | | 2 | 239 | 182 | 51 | 23 | 12 |
| Pirimiphos-ethyl | Insecticide, acaricide | 1 | 334 | 198 | 85 | 29 | 8 |
| | | 2 | 334 | 182 | 85 | 31 | 8 |
| Pirimiphos-methyl | Insecticide, acaricide | 1 | 306 | 108 | 71 | 43 | 6 |
| | | 2 | 306 | 67 | 71 | 65 | 6 |
| Pyriproxyfen | Insecticide | 1 | 322 | 96 | 56 | 21 | 4 |
| | | 2 | 322 | 78 | 56 | 81 | 2 |
| Prochloraz | Fungicide, herbicide | 1 | 376 | 308 | 16 | 17 | 13 |
| | | 2 | 376 | 266 | 16 | 23 | 13 |
| Profenofos | Insecticide, acaricide | 1 | 373 | 303 | 115 | 17 | 13 |
| | | 2 | 375 | 305 | 115 | 17 | 13 |
| Propiconazol | Fungicide | 1 | 342 | 159 | 46 | 37 | 13 |
| | | 2 | 342 | 89 | 86 | 99 | 4 |
| Propyzamide | Herbicide | 1 | 256 | 173 | 56 | 31 | 8 |
| | | 2 | 256 | 190 | 56 | 21 | 10 |
| Propoxur | Insecticide, acaricide | 1 | 210 | 168 | 58 | 11 | 24 |
| | | 2 | 210 | 93 | 58 | 33 | 7 |
| Prothioconazole-desthio | Fungicide | 1 | 312 | 70 | 76 | 61 | 2 |
| | | 2 | 312 | 125 | 76 | 49 | 6 |

| Pesticide | Functional class | Transitions | Precursor ion (m/z ±0.5) | Product ion (m/z ±0.5) | DP (V) | CE (V) | CXP (V) |
|---|---|---|---|---|---|---|---|
| Quinoxyfen | Fungicide | 1 | 308 | 197 | 61 | 43 | 7 |
| | | 2 | 308 | 162 | 61 | 57 | 7 |
| Rotenone | Insecticide, acaricide | 1 | 395 | 213 | 81 | 33 | 12 |
| | | 2 | 395 | 192 | 91 | 31 | 12 |
| Spinosad A | Insecticide | 1 | 732.5 | 142 | 51 | 37 | 13 |
| | | 2 | 732.5 | 98 | 51 | 75 | 13 |
| Spinosad D | Insecticide | 1 | 746.5 | 142 | 66 | 39 | 13 |
| | | 2 | 746.5 | 98 | 66 | 79 | 13 |
| Tebuconazol | Fungicide | 1 | 308 | 70 | 21 | 39 | 13 |
| | | 2 | 308 | 125 | 21 | 47 | 13 |
| Tebufenpyrad | Acaricide | 1 | 334 | 117 | 86 | 57 | 6 |
| | | 2 | 334 | 145 | 86 | 38 | 8 |
| Terbuthylazine | Herbicide | 1 | 232 | 176 | 81 | 25 | 7 |
| | | 2 | 230 | 104 | 81 | 45 | 6 |
| Teflubenzuron | Insecticide | 1 | 381 | 141 | 86 | 53 | 9 |
| | | 2 | 381 | 158 | 86 | 23 | 4 |
| Tetraconazole | Fungicide | 1 | 372 | 159 | 81 | 39 | 14 |
| | | 2 | 374 | 161 | 76 | 35 | 8 |
| Tetramethrin | Insecticide | 1 | 332 | 164 | 71 | 35 | 10 |
| | | 2 | 332 | 135 | 71 | 25 | 8 |
| Thiabendazole | Fungicide | 1 | 202 | 175 | 79 | 35 | 4 |
| | | 2 | 202 | 131 | 81 | 47 | 10 |
| Thiacloprid | Insecticide, moluscicide | 1 | 253 | 126 | 99 | 29 | 4 |
| | | 2 | 255 | 128 | 76 | 31 | 6 |

| Pesticide | Functional class | Transitions | Precursor ion (m/z ±0.5) | Product ion (m/z ±0.5) | DP (V) | CE (V) | CXP (V) |
|---|---|---|---|---|---|---|---|
| Thiamethoxam | Insecticide | 1 | 292 | 211 | 74 | 17 | 7 |
| | | 2 | 292 | 181 | 96 | 31 | 7 |
| Thiodicarb | Insecticide | 1 | 355 | 88 | 31 | 19 | 12 |
| | | 2 | 355 | 108 | 61 | 21 | 20 |
| Thiophanate-methyl | Fungicide | 1 | 343 | 151 | 76 | 23 | 14 |
| | | 2 | 343 | 93 | 81 | 71 | 6 |
| Tolclofos-methyl | Fungicide | 1 | 301 | 175 | 66 | 37 | 14 |
| | | 2 | 303 | 177 | 61 | 37 | 12 |
| Triadimefon | Fungicide | 1 | 294 | 225 | 61 | 19 | 14 |
| | | 2 | 296 | 199 | 36 | 21 | 12 |
| Triadimenol | Insecticide, fungicide | 1 | 296 | 70 | 41 | 37 | 12 |
| | | 2 | 296 | 227 | 11 | 15 | 13 |
| Triazophos | Insecticide, acaricide, nematicide | 1 | 314 | 119 | 76 | 47 | 6 |
| | | 2 | 314 | 286 | 76 | 10 | 24 |
| Trifloxystrobin | Fungicide | 1 | 409 | 186 | 59 | 23 | 7 |
| | | 2 | 409 | 145 | 116 | 63 | 8 |
| Triflumuron | Insecticide | 1 | 359 | 156 | 21 | 21 | 18 |
| | | 2 | 359 | 139 | 21 | 45 | 18 |
| Tricyclazole | Fungicide | 1 | 190 | 163 | 89 | 31 | 7 |
| | | 2 | 190 | 136 | 91 | 36 | 7 |
| Zoxamide | Fungicide | 1 | 336 | 187 | 46 | 31 | 24 |
| | | 2 | 336 | 159 | 46 | 55 | 20 |

DP- Declustering potential (V); CE- Collision energy (V); CXP- Collision cell exist potential (V); DMST- N,N-dimethyl-N'-p-tolysulphamide; EPN- O-ethyl O-4-nitrophenyl phosphonothiate

**Table S2.** Parameters for determination of pesticides residues in rice by HPLC-MS/MS in ESI- mode. Transition 1: Quantification transition; Transition 2: Confirmation transition.

| Pesticide | Functional class | Transitions | Percursor ion (m/z ±0.5) | Product ion (m/z ±0.5) | DP (V) | CE (V) | CXP (V) |
|---|---|---|---|---|---|---|---|
| **DNC** | | | 301 | 137 | -120 | -16 | -15 |
| **Fludioxonil** | Fungicide | Transition 1 | 247 | 180 | -65 | -40 | -9 |
| | | Transition 2 | 247 | 126 | -65 | -42 | -7 |
| **Fipronil** | Insecticide | 1 | 435 | 330 | -60 | -20 | -4 |
| | | 2 | 437 | 332 | -60 | -20 | -4 |
| **Methoxyfenozide** | Insecticide | 1 | 367 | 149 | -142 | -17 | -16 |
| | | 2 | 367 | 105 | -142 | -29 | -16 |

DP- Declustering potential (V); CE- Collision energy (V); CXP- Collision cell exist potential (V)
